# The use of smartphone in measuring stance and gait patterns in patients with orthostatic tremor

**DOI:** 10.1371/journal.pone.0220012

**Published:** 2019-07-18

**Authors:** Jung Hung Chien, Diego Torres-Russotto, Zhuo Wang, Chenfan Gui, David Whitney, Ka-Chun Siu

**Affiliations:** 1 Physical Therapy Education, College of Allied Health Professions, University of Nebraska Medical Center, Omaha, Nebraska, United States of America; 2 Department of Neurological Sciences, College of Medicine, University of Nebraska Medical Center, Omaha, Nebraska, United States of America; Universidade Estadual Paulista Julio de Mesquita Filho, BRAZIL

## Abstract

Orthostatic tremor (OT) is a rare movement disorder characterized by a fast tremor (13–18 Hz) in the lower extremities during stance. Patients with OT typically complain of instability while standing/walking. However, due to the geographical limitation, the standing instability or gait problems in patients with OT cannot be assessed and monitored frequently. The increasing popularity of using smartphone-based accelerometers could be a solution to eliminate this limitation. This study examined the feasibility of using smartphone-based accelerometers to identify the changes in body movement in different standing and locomotor tasks. Twenty patients with OT and seven healthy controls were consented to participate in this study. Subjects stood with eyes open or eyes closed for 20 seconds. They also performed four different locomotor tasks (normal walking, tandem walk, walking on an elevated surface, and obstacle negotiation). When performed different locomotor tasks, patients with OT had a larger acceleration of body movement than controls in the medial-lateral direction (tandem walk: *p* = 0.026, walking on an elevated surface: *p* = 0.002, and stepping over the obstacle: *p* = 0.028). Patients with OT had smaller acceleration of body movement than controls while standing with eyes open in the vertical direction (*p* = 0.012), in the anterior-posterior direction (*p* = 0.013) and in the medial-lateral direction (*p* = 0.011). This study provides objective evidence of balance instability in patients with OT not only while standing but also during different challenging locomotor tasks by using smartphone-based accelerometers.

## Introduction

To maintain proper standing balance or gait progression in human requires a series of sensory processing and motor executing [1]. Sensory input from the environment provides information to the brain [1]. The human brain organizes, integrates, synthesizes, and uses this specific information to organize and execute the motor units to generate appropriate responses [1]. If any of the sensory systems is deteriorated, the different adaptation of stand/gait pattern is observed [2–3]. Thus, to assess standing stability/gait pattern can be used to identify the different neurological disorders such as hemispastic gait, paraspastic gait, ataxic gait, or freezing gait [4–7]. However, most of these gait patterns are typically assessed in clinics or research laboratories. For patients with gait disorders and live in rural areas far from cities, to routinely assess or monitor their gait patterns becomes inaccessible. Therefore, there is a special need to develop an objective and accessible way to enable periodically quantitative assessment of body movement (e.g., gait) in these patients with gait disorders living in long distance from clinical or research facilities.

The smartphone, which contains the touch screen function and 3-dimensional accelerometers similar in the sensitivity to research-grade biomechanical instrumentation, is a perfect solution to fulfill the special need. Emerging studies have developed and validated different mobile applications by using touch screen function to detect the automated decision in cognitive tasks in patients with Huntington Diseases, Alzheimer, and Parkinson disease [8]. Also, the smartphone-based accelerators have been used to detect gait events (e.g., heel-strike, and toe-off) [9], as well as the differences in acceleration signals between patients with and without gait disorders, such as patients with Parkinson’s disease [10]. In the latest study, the accelerometers are used to identify the subtle gait differences during normal and dual-task walking in older adults [11]. These studies use the filtered acceleration data (Butterworth filter, 4th order, zero lag) to estimate the moment of heel contact and toe-off by finding the peak values during the gait cycle [9–11]. Also, a study not only investigates the time domain of acceleration data but also convert the time domain of acceleration data into the frequency domain by using Fourier Transformation to identify the moment of heel contact and toe-off accurately [12]. In addition, to provide security and theft prevention, these smartphone-based accelerators are used to identify user’s confidential identification for unlocking the smartphone as a biometric parameter by measuring their changes of acceleration in their gait characteristics [13]. The model of this previous study used is the Random Projections and probability density function as a decision function to predict the gait characteristics [13]. The abovementioned studies have shown a wide range of possible solutions to recognize the gait characteristics using smartphone-based acceleration data. Thus, in the current study, we used these validated accelerometers to detect the stance and gait patterns in patients with orthostatic tremor (OT) by using the frequency domain analysis.

Orthostatic Tremor is a rare neurologic disease (one OT patient in a cohort of approximately 4000 elderly subjects in Spain [14]) characterized by a fast tremor (13–18 Hz) in the lower extremities leading to a strong sensation of falling during stance [15–18]. The etiology and pathophysiology of OT remain unclear, but localization seems to point to the posterior fossa [14]. Common validated balance measurements are significantly abnormal in OT while standing [15], indicating that the center of pressure in patients with OT moves faster than healthy controls in the anterior-posterior direction [16]. Other studies show differences in the spectral power density of the center of pressure trajectory (the peak of spectral power density appears in the higher frequency region, indicating higher alterations of body acceleration) in patients with OT while standing with eyes closed [17–18]. These studies seem to agree in that symptoms of OT (tremors in lower legs) force patients altering the body acceleration to compensate the abnormal somatosensory system [16–18]. These OT symptoms can be alleviated by sitting or walking [11, 19–21]. However, the latest study provides an alternative finding that tremor intensity is phase-dependently modulated [22] during walking. Specifically, only during the stance phase of the gait cycle, high frequency of leg muscles activation (high tremor intensity) is observed in these OT patients, resulting in higher stride-to-stride fluctuations of gait parameters [22]–an indication of gait instability [23] when walking on a treadmill. In fact, due to the increasingly compromised gait pattern, 25% of falls in patients with OT is reported from medical record [24]. However, the aforementioned studies only focus on the regular level of walking. For real life, to perform a tandem walk when walking in the narrow-based alley, to walk on the elevated surface, and to step over an obstacle are all parts of daily activities. To investigate these locomotor tasks might better assist patients with OT to reduce the potential risks of falls.

Our medical center is one of the few institutions to organize annual international conferences and to conduct gait assessments in patients with OT. In general, gait assessment for these patients might only be evaluated objectively on an annual basis, and limited gait information based on the progression of this disease could be objectively obtained. Therefore, our goal is to increase the accessibility for those patients to have a routine evaluation on the body movement and to deepen our current knowledge on how standing balance and gait changes in patients with OT by using smartphone-based accelerometers. The dependent variable was the mean frequencies of power spectral density of accelerations (PSDA) [17–18]. This study hypothesized that patients with OT would increase the mean frequency of PSDA in all three directions (vertical, medial-lateral, and anterior-posterior) in comparison with healthy controls while standing with eyes open and eyes closed. In addition, we hypothesized that patients with OT would increase the mean frequency of PSDA in three directions in comparison with controls while performing different locomotor tasks.

## Materials and methods

### Subjects

This study was performed as part of the longitudinal Orthostatic Tremor Research Study in our medical center. Patients have been recruited through support groups and social media from the USA, Canada, Europe, and Australia. All patients were native English speakers. A subset of participants in the longitudinal study was invited to participate in this study. Twenty-seven participants were enrolled in this study including seven healthy controls (2 females and 5 males, average age: 75 ± 10.05 years old; average height: 1.69 ± 0.09 m; average body mass: 72.96 ± 9.99 kg) and 20 patients with OT (18 females and 2 males, average age: 67.95±7.30 years old; average height: 1.65 ± 0.06 m; average body mass: 76.33 ± 15.98 kg; Table 1). More information was provided in Table 1. None of the participants experienced any falls during the previous year. Participants in the healthy control group did not have any history of neurological disease and had no family history of tremor and other movement disorders. Healthy participants were recruited from the accompanies of OT patients and from the employees of our medical center with age- and body mass-matched to patients. All 20 patients fulfilled the Movement Disorders Society’s criteria for OT, including the presence of a 13-18Hz tremor in the lower extremities while standing, as seen in surface electromyography (EMG) [25]. In this study, only patients with primary OT without an associated postural arm tremor were recruited based on the EMG screening. In addition, if patients had OT and additional neurological features, such as dementia, essential tremor, ataxia, and Parkinson’s disease, were excluded to reduce the confounding factors for measuring the balance control in standing or walking (Table 1). Patients were also excluded from the study if they had a history of visual or vestibular deficits or any neurological disorders other than OT. We verbally asked subjects “Can you see your surroundings clearly when you walk straight without glasses.” If subjects answered negatively, visual acuity was corrected by wearing glasses. Patients were asked to withhold medications used to treat OT overnight prior to the study in order to reduce the effect of medication on gait pattern. All participants were living independently and did not require a walking aid (cane or walker) during the testing session.

**Table 1:** Characteristics of Patients with Orthostatic Tremor (OT). Duration of Disease is the years from first diagnosis of OT to the date of data collection. The highlighted patients are those who completed all standing and locomotor tasks.

Patients	Gender	Age (years)	Height (m)	Weight (kg)	Duration of Disease (years)
1	M	66	1.75	79.37	20
2	F	76	1.60	52.61	21
3	F	61	1.70	72.57	10
4	F	76	1.67	77.11	13
5	F	78	1.62	70.30	11
6	F	63	1.64	61.23	21
7	F	60	1.67	90.71	15
8	F	60	1.67	110.67	3
9	F	61	1.62	68.03	15
10	F	68	1.70	83.91	18
11	F	52	1.58	51.25	9
12	F	57	1.65	75.74	11
13	F	72	1.70	88.45	10
14	F	65	1.70	65.77	20
15	F	68	1.79	84.82	20
16	F	74	1.55	102.96	21
17	F	70	1.68	58.96	21
18	F	70	1.68	79.37	6
19	F	75	1.65	68.03	12
20	F	78	1.61	79.83	15

All participants also filled the Falls Efficacy Scale (FES) [26] questionnaire which is widely validated and designed to assess fear of falling. This FES contains 10 questions to answer how confident patients are to do the following activities without falling (with 1 being very confident and 10 being not confident at all): Take a bath or shower, reach into cabinets or closets, walk around the house, prepare meals not requiring carrying heavy or hot objects, get in and out of bed, answer the door or telephone, get in and out of chair, getting dressed and undressed, personal grooming, and getting on and off of the toilet [27]. A total score greater than 70 stronger indicates that this person has a strong sensation of the fear of falling [27]. This study was carried out in accordance with the relevant guidelines and regulations of and upon approval by our Institutional Review Board (IRB# 389-12-EP). Informed consent was obtained from all participants before the experiments began.

### Experimental materials

**Fig 1:**
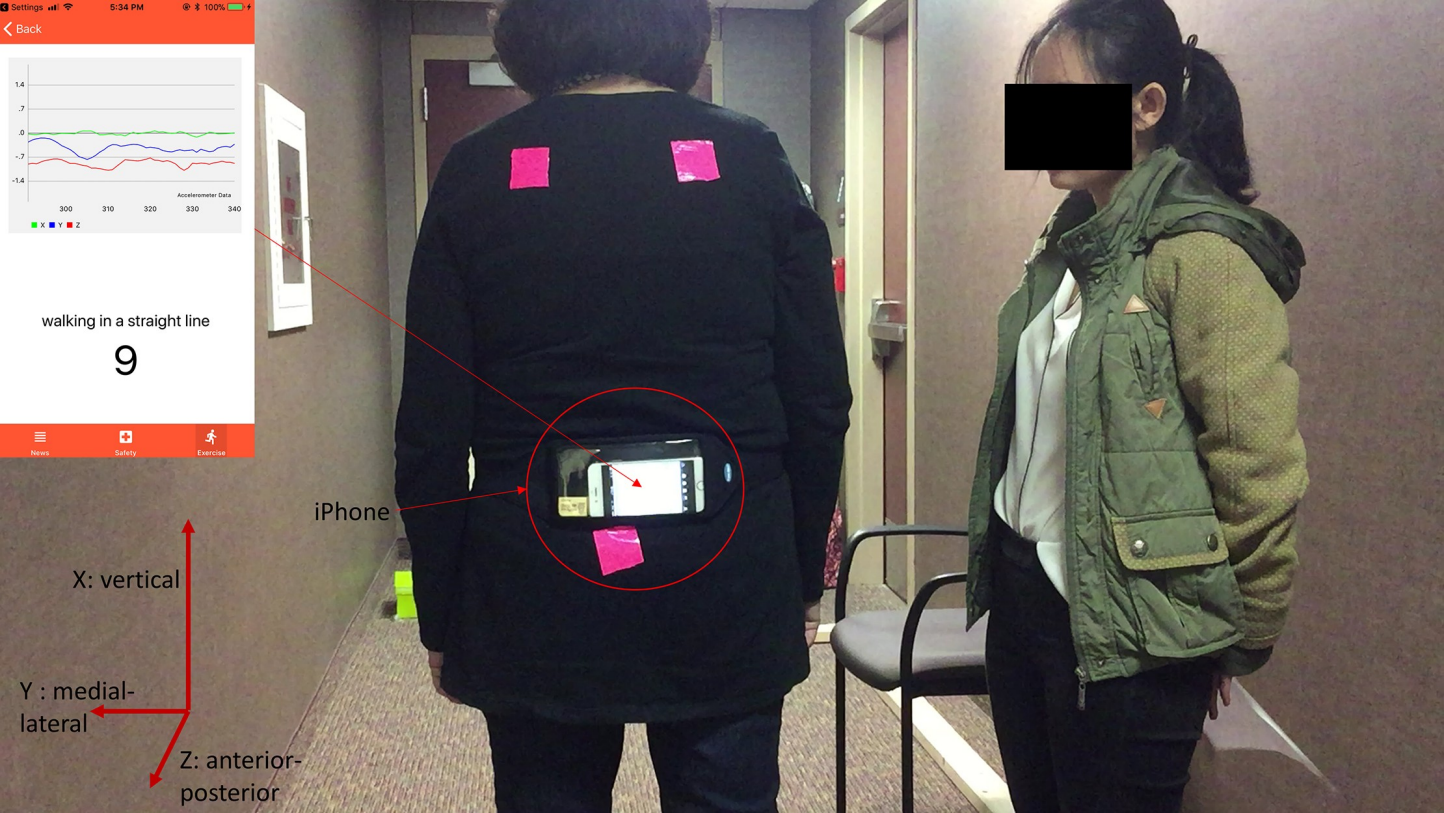
The smartphone application structural diagram.

**Fig 2:**
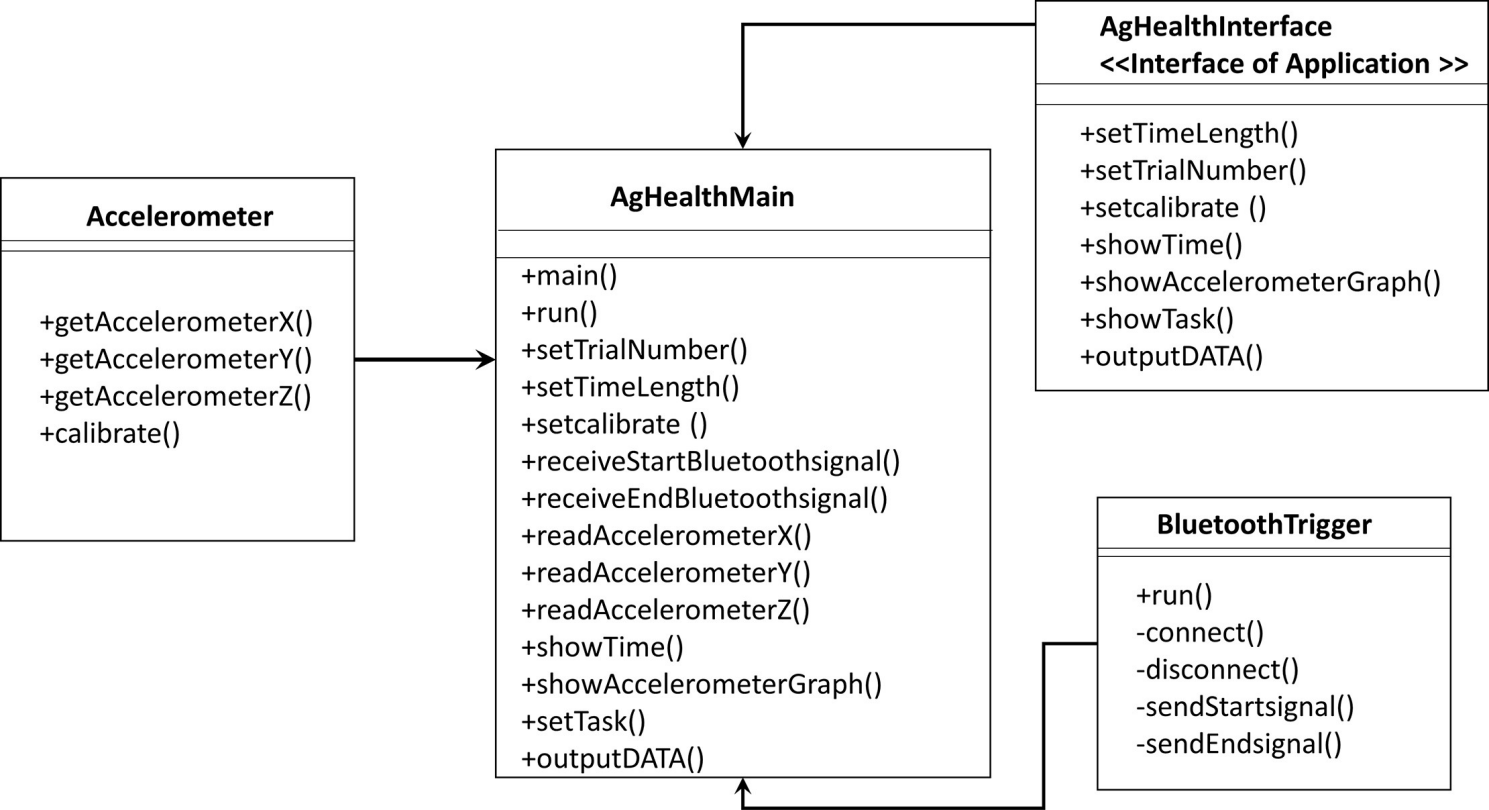
The smartphone application UML activity diagram.

**Fig 3:**
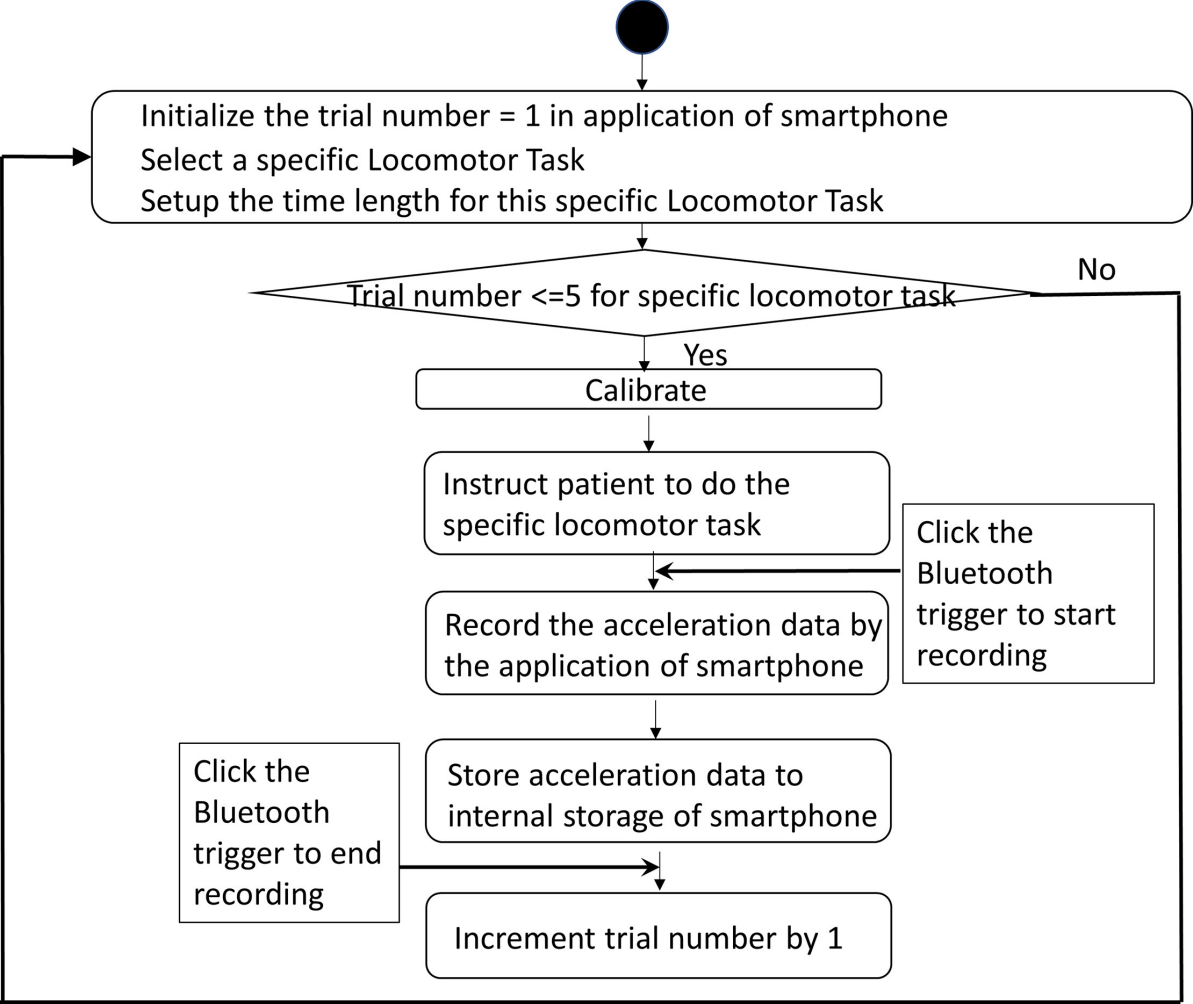
Multi-directional accelerometry using an iPhone. The smartphone application is seen in the left-top corner. Green line: the acceleration in the vertical direction, Blue line: the acceleration in the medial-lateral direction. Red line: the acceleration in the anterior-posterior direction.

A smartphone (iPhone 6s, iOS 11.2.5, Apple Inc.) was used to quantify the stand/gait pattern by measuring the accelerations in different directions [28–29]. Also, this smartphone was fastened tightly on the sacrum area via running belt waist pack to reduce the noise from the unexpected movement of the smartphone (Fig 1). The sacrum area has been indicated as the location of the center of mass of the body, where is the center of balance control [30]. This smartphone application was written via Xcode (Apple Inc) by using embedded three accelerometers of the iPhone to quantify the balance. The structural diagram and UML activity diagram were shown in Figs 2 and 3. The sample rate was at 50Hz, and acceleration values for each axis were reported directly by the smartphone as G-force values. One trigger connected to a smartphone through Bluetooth allowed the research team to click start and end point of each test. In this study, all data were saved in the iPhone local memory system.

### Experimental procedure

**Fig 4:**
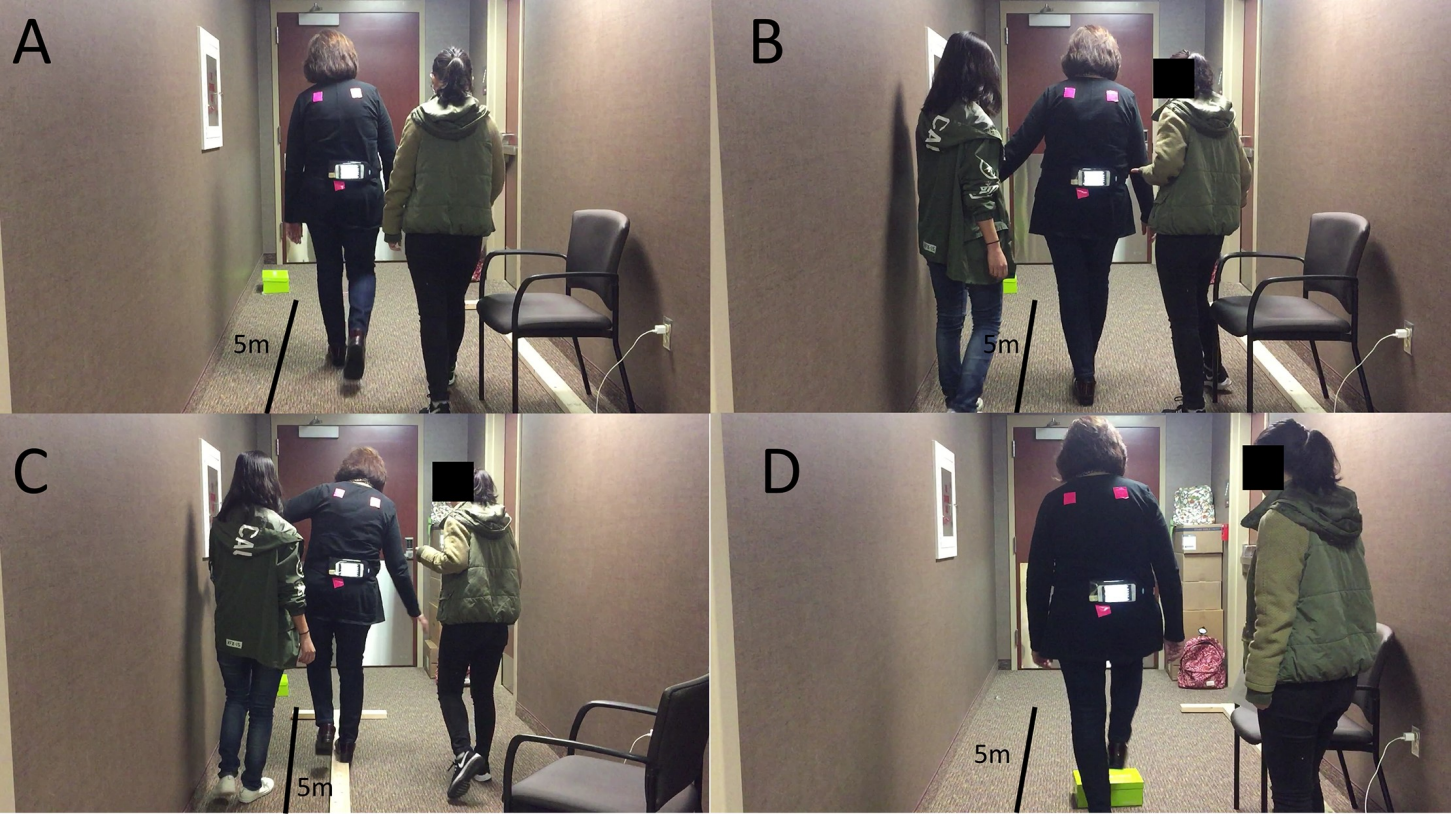
Four different locomotor tasks–(A) Normal walking; (B) Tandem walk; (C): T-bar walk; (D) Obstacle negotiation.

All participants were instructed to complete two conditions: standing and walking. In the standing condition, subjects needed to stand with eyes open for 20 seconds and with eyes closed for another 20 seconds. Subjects needed to perform 4 activities (Fig 4): 1) walking straight for 500-centimeter distance, 2) tandem walk (where the toes of the back foot touch the heel of the front foot at each step) for 500 centimeters, 3) T-bar walk (walking on an elevated T-bar: length: 500 cm, width: 10 cm, and height: 4cm), and 4) obstacle negotiation (walk straight for 500-centimeter and stepping over the shoe box which is located at 200-centimeter from the starting point, the dimension of the show box: length: 29 cm, width: 19 cm, height: 10.5 cm). In both conditions, each task was performed 3 times. Subjects were asked to sit and rest between each task as long as they needed. To prevent falls, two team members always stood or walked along with participants without any interference (Fig 4).

### Data reduction

All acceleration data were sampled at 50Hz. The mean frequencies of power spectral density of accelerations (PSDA) in three axes, as dependent variables, during standing and walking were computed through MATLAB R2011a program (Mathworks, Natick, MA). For deriving the frequency domain features, Fourier transformation and power spectral density were used. The power spectral density gave a variation of signal power versus frequency while the Fourier transform gave the variation of signal magnitude versus frequency (see equation below) [31].

$${PSDA}_{x}(j\omega )=F[R_{xx}(\tau )]=\int_{-\infty }^{\infty }R_{xx}(\tau )e^{-j\omega t}d\tau$$

where, *PSDA_x_*(*jω*) was the power spectral density of the process, x, F [] indicated Fourier transform, and *R_xx_*(*τ*) is the autocorrelation of the process, x. The mean frequency was derived from the below equation.

$$mean\;of\;PSDA=\frac{\left(fs\;\sum PSDA\right)}{Length\;of\;PSDA}$$

where *f*_*s*_ is the sampling frequency of the signal, which is 50Hz in the current study, and the length of PSDA is the number of the points for which PSDA was calculated [32].

The measure of the mean frequency of PSDA has been used to detect the subtle abrupt changes in a series of movements [17–18]. In the current study, larger mean frequency of PSDA indicated that higher body sway acceleration (the faster alternation of body sway velocity) was observed to adapt to the environment and vice versa. However, either too high or too low mean frequency of PSDA indicated a compromised gait/stand pattern [33]. Also, the average gait speeds in four different locomotor tasks and FES score are other dependent variables. The average gait speed was calculated as walking distance / completed one locomotor task time. Out of the 20 patients, only seven were able to complete all tasks. Eight out of 20 OT patients decided not to perform the tandem walk. Also, eleven out of 20 OT patients decided not to walk on the T-bar. Therefore, we only used the data from those seven patients for data analysis based on the suggestion from our biostatistician.

### Statistical analysis

For standing condition, a mixed three-way repeated ANCOVA was used (2 groups x 2 visual conditions x 3 directions, with the duration of OT diagnosed as a covariate) was used to investigate the interaction among the effect of OT, the effect of the vision, and the effect of direction. For walking condition, a mixed three-way repeated ANCOVA (2 groups x 4 tasks x 3 directions, with the duration of OT diagnosed as a covariate) was used to investigate the interaction among the effect of group, the effect of different walking tasks, and the effect of different directions for each dependable variable during walking. The duration of OT diagnosed was set as four categories: normal, 1–10 years, 11–20 years, and above 20 years. When a significant interaction was determined, the post-hoc pairwise comparisons were used. Statistical analysis was completed by using SPSS 18.0 (IBM Corporation, Armond, NY). The significance level was set at 0.05. In order to understand the effect of duration (from the time has been diagnosed for primary OT to the date attended the data collection) on making decision whether to perform locomotor tasks or not, an independent t-test was performed between patients in OT who completed all tasks, and patients in OT who did not complete all tasks. Also, a non-parametric Kruskal Wallis test was used to compare the sensation of the fear of falling (FES score) among controls, patients in OT who completed all tasks, and patients in OT who did not complete all tasks. The pairwise comparisons were used if there was an asymptotic significance among these three groups. There a few reasons to select a mixed three-way repeated ANCOVA in the current study were as followings: 1) all subjects completed every standing and walking conditions (with/without vision in standing or four different locomotor tasks in walking); therefore, the repeated measure method was used; 2) to understand the differences of PSDA between groups (OT/controls), the mixed method was used; and 3) to understand whether the duration of OT diagnosed was a confounding factor and all abovementioned investigations, an ANCOVA was applied. Also, a non-parametric Kruskal Wallis test was used to identify the effect of fear of falling sensation on controls and patients with OT who didn’t complete all of the standing and walking tasks. Due to the different sample size between groups, a non-parametric Kruskal Wallis test was used. To examine the effect size, we used the partial eta squared method. The partial eta squared has been commonly used for measuring the effect size and was at least 0.138 for large effect size, 0.059 for moderate effect size, and 0.01 for small effect size [34]. All statistical analyses were conducted by our biostatistician.

## Results

No fall incident was observed in this study in both OT and control groups. However, only seven OT patients (age: 63.86 ± 7.63 years old; height: 1.65 ± 0.05 m; body mass: 74.84 ± 14.65 kg) completed all tasks in both standing and walking conditions.

### For standing condition (Table 2)

A significant interaction among the effect of the vision, the effect of OT, and the effect of direction on the mean frequency of PSDA were found (F_2,22_ = 22.454, *p* < 0.0001); however, there was no effect of duration on the mean frequency of PSDA. The post hoc results indicated that the mean frequency of PSDA was significantly lower in patients with OT than controls in the vertical direction (*p* = 0.012), in the anterior-posterior direction (*p* = 0.013), in the medial-lateral direction (*p* = 0.011) when vision was available during standing. However, when vision was absent, the mean frequency of PSDA was significantly higher in patients with OT than controls in the vertical direction (*p* = 0.022), in the anterior-posterior direction (*p* = 0.012), in the medial-lateral direction (*p* = 0.038).

**Table 2:** The mean frequency (SD) of power spectral density of accelerations (PSDA) in three different directions during different standing conditions.

Standing	Eyes-Open	Eyes-Open	Eye-Closed	Eyes-Closed
Groups	Control	OT	Control	OT
**Mean frequency of PSDA in Vertical Direction (Hz)**	6.82(1.17)	5.18(0.87)*	7.15(1.83)!	11.29(2.78)*!
**Mean frequency of PSDA in Anterior-Posterior Direction (Hz)**	7.75(1.05)	4.98(0.59)*	8.15(1.49)	11.38(2.27)*!
**Mean frequency of PSDA in Medial-lateral Direction (Hz)**	7.74(1.32)	5.75(1.24)*	8.53(1.15)	10.65(2.11)*!

*:represents significant differences between patients with OT and Controls.

!: represents differences between Eyes-Open and Eyes-Closed.

### For walking and other locomotor movements (Table 3)

There was no significant interaction between the effect of OT and the effect of different locomotor tasks on walking speed. However, a significant interaction among the effect of walking task, the effect of group, the effect of direction on the mean frequency of PSDA was found (F_6, 66_ = 5.232, *p* < 0.0001). There was no effect of duration on the mean frequency of PSDA. The post hoc comparisons indicated that the mean frequency of PSDA was significantly higher in patients with OT than controls when performing tandem walk (p = 0.026), T-bar walk (p = 0.002), and obstacle negotiation (p = 0.028) in the medial-lateral direction.

**Table 3:** The mean frequency (SD) of power spectral density of accelerations (PSDA) in three different directions during different walking conditions.

Walking	Normal Walking		Tandem Walk		T-bar		Obstacle Negotiation	
Group	Control	OT	Control	OT	Control	OT	Control	OT
**Walking Speed (m/s)**	1.08(0.13)	1.29(0.21)	0.51(0.16)	0.44(0.18)	0.70(0.11)	0.67(0.25)	0.88(0.15)	0.88(0.13)
**Mean frequency of PSDA in Vertical Direction (Hz)**	4.91(0.85)	5.18(0.87)	5.82(0.77)&	5.86(0.46)&	5.68(0.82)	5.53(0.83)	5.90(0.99)	5.32(0.84)
**Mean frequency of PSDA in Anterior-Posterior Direction (Hz)**	5.81(0.74)	5.75(1.24)	5.43(0.92)	5.34(0.63)	5.32(0.45)	5.69(0.66)	5.65(0.35)	5.71(0.45)
**Mean frequency of PSDA in Medial-lateral Direction (Hz)**	5.18(0.98)	4.98(0.59)	5.17(2.05)	7.26(0.72)*&	5.46(0.81)	6.72(1.04)*&	4.48(1.32)	6.22(1.60)*&

*: represents significant differences between patients with OT and Controls.

&: represents differences between Tandem walk, T-bar or Obstacle negotiation and Normal walking condition.

### The effect of the duration in patients of OT on task completion

There was no statistically significant effect of duration in patients of OT on task completion.

### The effect of the sensation of the fear of falling on task completion (Fig 5)

A non-parametric Kruskal Wallis test showed a significant effect of the sensation of the fear of falling on task completion (F_2,24_ = 5.56; *p* = 0.01). Multiple comparisons indicated that the FES in patients who did not complete tasks was significantly higher than in those who completed all tasks (*p =* 0.029). Also, the FES in patients who did not complete tasks was significantly higher than in controls (*p* = 0.005). There was no significant difference between the FES between controls and patients who completed all tasks.

**Fig 5:**
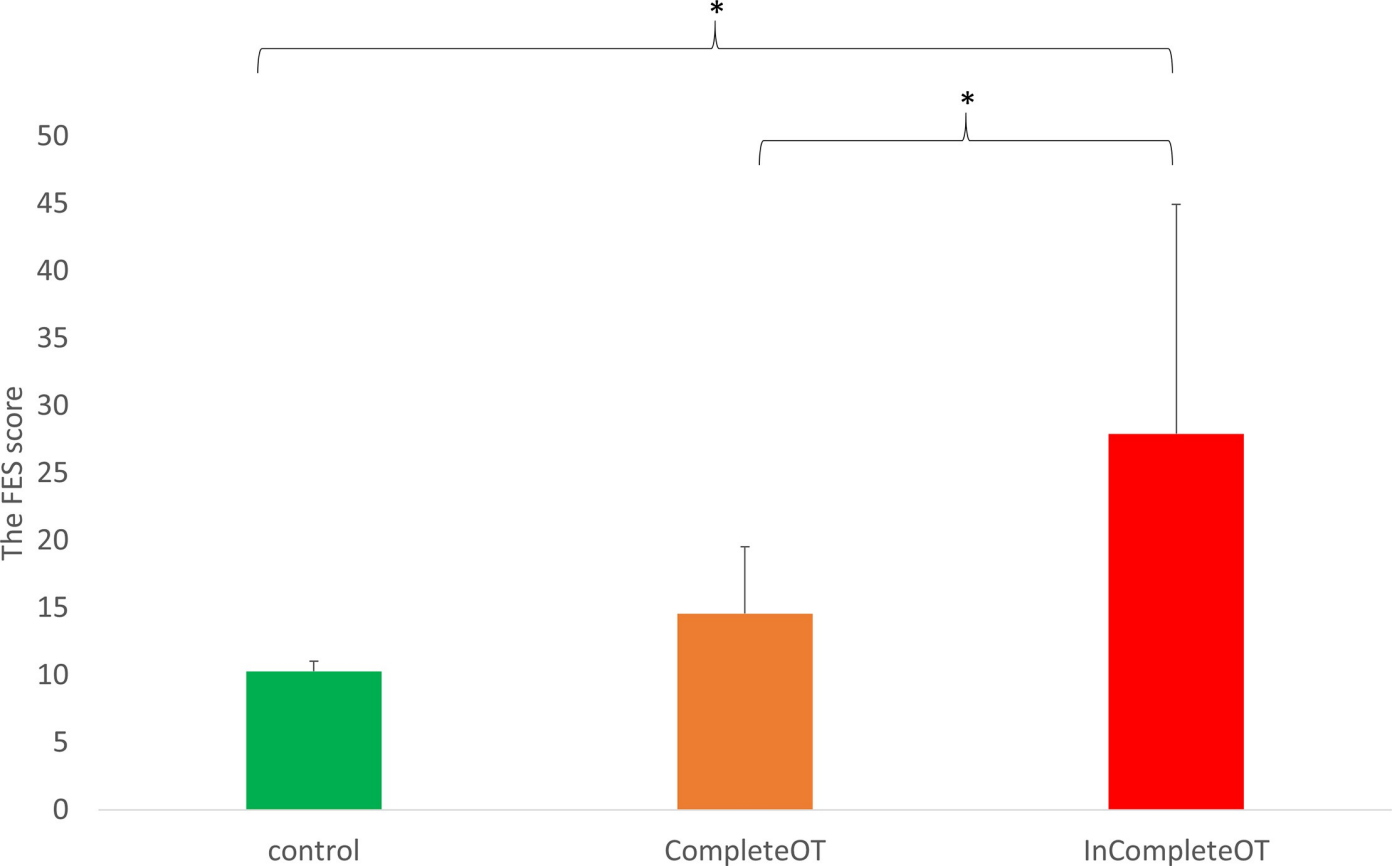
The effect of FES score on task completion. CompleteOT: patients who complete all standing and locomotor tasks. InCompleteOT: patients who were absent any either standing or locomotor tasks.

### The effect size

For the significant interaction among the effect of direction, the effect of the vision, and the effect of OT, the partial eta squared was 0.613 in a standing condition. For the significant interaction among the effect of direction, the effect of different locomotor tasks, and the effect of OT, the partial eta squared was 0.322 in walking condition. These results indicated that the effect size was large [34].

## Discussion

This study confirmed our hypothesis that using smartphone-based accelerometers could identify the changes in the standing balance/gait patterns. The overall results were that even if patients with OT showed no difference in comparison with controls in normal walking, they still demonstrated different balance control while performing complex locomotor tasks and standing without visual support.

In our current study, larger mean frequency of PSDA in the medial-lateral direction was found in patients with OT while performing more challenging walking tasks. It is commonly observed that while performing a tandem walk or walking on a narrow and elevated pathway, participants might reduce their walking speed to ensure safety and reduce fall incidence. They would also frequently change their instantaneous velocities (swaying back and forth laterally and continuously) in the medial-lateral direction to actively control their balance. It can be speculated that patients with OT significantly increased their accelerations in the medial-lateral direction than healthy controls due to the loss of sensitivity to the positions from proprioceptive information in patients with OT [22]. This loss of sensitivity of proprioceptive information might lead to the increment of double support time [20], which is one of major indicators for fallers [35]. Therefore, these results suggested that patients of OT might have a higher risk of falling in some daily activities in comparison with normal individuals, even though they do not have any fall history.

Also, the results found no significant difference in accelerations in all directions between patients with OT and healthy controls while walking normally. For our observation, it has been shown that there was no balance concern during normal walking in patients with OT although the abnormal muscle activations are found [22]. However, even these patients can walk normally; several studies indicate that the quality of life is severely affected in OT due to the limitations of activities and social life [36–37]. Therefore, it can be speculated that the psychological status (e.g., the fear of falling) might be a major factor to impact the quality of life in patients with OT. While standing, multiple sensory feedback loops to the spinal cord, brainstem, cerebellum, thalamus, and motor cortex are active during controlling the head and body orientation. It is possible that malfunction of proprioceptive afferents from muscle spindles in patients with OT increase the excitability in the cerebellum, thalamus, and prefrontal cortex through the ponto-cerebello-thalamo-cortical pathway [38]. In addition, deactivations in medial frontal areas in patients with OT has an impact on postural control via primary motor cortex [38] by influencing the controls of emotional responses (e.g., fear) and decision making [39]. This could also explain the fact that more than half of patients with OT in this study were unable to perform the challenging walking tasks. In order to verify this phenomenon, the FES was used to understand the effect of the sensation of the fear of falling on the completion of the tasks. Our results confirmed that patients who did not complete the balance or locomotor tasks had higher FES score than patients who completed the study entirely. Psychological impact in patients with OT should require additional attention in the clinical evaluation.

Surprisingly, our results did not support our hypothesis that the mean frequency of PSDA reduced significantly in patients with OT than in healthy controls when the vision was available in standing. Possibly as sensory conflicts increased while standing (non-stop “shaking” legs–tremulous disruption of proprioceptive input), patients with OT did not have many options for balance control but limited the body accelerations to maintain their balance through the visual control. It is likely that patients with OT attempted to control their body sway acceleration very hard through their vision in order not to fall when their proprioceptive inputs were tremulously disrupted. This speculation is similar to a previous study when young, and older adults stand on the rotating platform (perturbed proprioceptive system environment), they freeze their degree of freedom of standing movement to maintain balance [40].

It was a different scenario when the vision system was not available. When both visual and proprioceptive sensory systems became unreliable or unavailable, patients with OT demonstrated faster sway velocity changes in comparison with healthy controls in the current study. The faster changes in velocity and higher velocity of body sway might be explained by Bayesian framework [41]. The Bayesian framework indicates that sensory ambiguity leads to a broader probability curve of postural sway estimation and uncertainty regarding necessary postural corrections when a single modality is involved^36^. When an additional sensory signal is available, the integrated signal leads to a more precise estimation and subsequently more appropriate postural corrections [41]. Therefore, in the current study, when both vision and somatosensory systems became unreliable or unavailable, the balance control was impacted in patients with OT than in healthy controls.

In summary, this study verified that using smartphone-based accelerometers can identify the standing balance and gait changes in patients with OT. However, there were still several limitations to this study. The first limitation was to only half of the patients with OT completed all locomotor tasks; the sample size was limited to draw a generalizable conclusion. However, our biostatistician has used the optimal statistical analysis to control the limitation and compare the results between patients and controls. In addition, the size effect was large based on partial eta squared analysis; therefore, the power of this study and the use of a statistical model was verified. Secondly, we cannot know which momentary state the patients are or instantaneous changes during walking because we did not use the traditional motion capture system. However, we used the spectrum analysis to investigate the overall gait pattern between patients with OT and controls. The spectrum analysis was sensitive to detect the abnormal movement in the overall pattern. Thirdly, the standard deviation of body mass in both controls and OT patients were high. As mentioned above, OT is a rare disease. Therefore, it is apparently a limitation to control the subjects’ inclusion criteria based on body mass. In Table 1, the body mass of our OT patients ranged from 60 to 100 kg. To overcome this limitation, age-matched and weight-matched controls were recruited in this study.

## Supporting information

S1 File Data file. (XLSX)
